# Using Sorbitol as Electrolyte Additive to Control Interfacial Environments in Electrochemical CO_2_ Reduction on Silver

**DOI:** 10.1021/acscatal.5c04382

**Published:** 2025-09-16

**Authors:** Anil Kumar Sihag, Florian Altmann, Alper T. Celebi, Markus Valtiner, Christian M. Pichler

**Affiliations:** † Institute of Applied Physics, 27259 Vienna University of Technology, 1040 Vienna, Austria; ‡ Center for Electrochemical Surface Technology, 2700 Wiener Neustadt, Austria

**Keywords:** CO_2_ electroreduction, Sorbitol, Molecular dynamics simulations, Flow cell, Silver, Gas diffusion electrodes

## Abstract

The utilization of electrolyte additives in the electrochemical reduction of CO_2_ (CO_2_RR) is an effective way to attenuate the hydrogen evolution reaction and increase the yield of carbon-based products. Frequently, the beneficial additive effects can only be demonstrated in batch cell experiments, and the molecular mechanisms behind the observed effects are unclear. Herein, we investigate the impact of sorbitol as an electrolyte additive in the CO_2_RR using practically relevant gas diffusion electrodes (GDE) with silver nanoparticles as catalyst, while gaining insight into the reaction mechanisms by molecular dynamics (MD) simulations. The addition of 100 mM sorbitol to the aqueous electrolyte resulted in a notable enhancement of the CO faradaic efficiency, rising from 79% to 90%, while concurrently minimizing the hydrogen evolution reaction (HER) from 15% to 5% at a current density of 98 mA·cm^–2^. MD simulations were employed to investigate changes in the electrode/electrolyte interface composition. The addition of sorbitol resulted in a decrease in the concentrations of bicarbonate ions near the electrode interface, while no significant variations were observed for water and CO_2_ concentrations. Stronger coordination was observed between potassium ions and CO_2_ molecules due to the presence of sorbitol. This interaction may stabilize the CO intermediates and enhance the CO_2_RR efficiency. As bicarbonate can be an important intermediate for HER, its reduced concentration is benefiting CO_2_RR. This study is a rare example of how a nonionic electrolyte additive such as sorbitol can influence the structure of the solid/liquid interface in electrocatalysis and enhance the performance in CO_2_RR.

## Graphical abstract

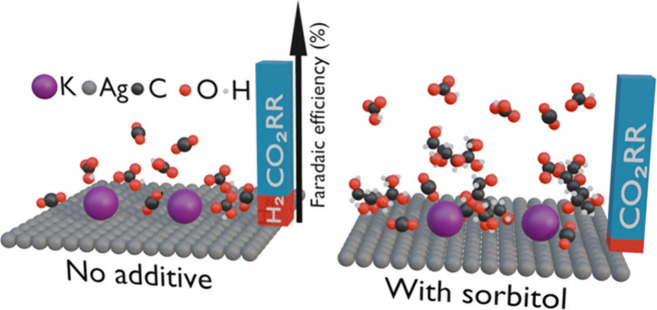

## 1 Introduction

The electrochemical CO_2_ reduction reaction (CO_2_RR) presents a promising avenue for transforming CO_2_ into valuable chemicals and fuels, avoiding the utilization of fossil feedstocks.^1,2^ Despite the activity of numerous metal catalysts for CO_2_ reduction reaction (CO_2_RR), in aqueous electrolytes, most of these catalysts suffer from hydrogen evolution reaction (HER) as a side reaction, thereby limiting the actual CO_2_ conversion.^3–8^ Previous studies have demonstrated how the composition of catalyst surface and solid/liquid interfaces can result in improved catalytic performance.^9,10^ Therefore, the modification of the local environment at the electrode–electrolyte interface has emerged as a promising strategy to mitigate HER and enhance CO_2_RR activity.^6,11–15^ In this context, the use of pure ionic liquids (ILs) or ILs as additives in aqueous electrolytes has been extensively explored to suppress side reactions and improve CO_2_RR efficiency.^16^ Feaster et al. reported that low concentrations of ionic liquid 1-ethyl-3-methylimidazolium chloride ([EMIM]­Cl) can suppress the HER in acidic electrolytes using several transition metal electrodes, whereas barely no HER suppression was observed in basic media.^17^ This effect was attributed to the displacement of interfacial H_3_O^+^ ions in the acidic medium by [EMIM]^+^ cations, while the interfacial neutral H_2_O molecules remained unaffected in the alkaline medium.

Two primary hypotheses are proposed to explain the suppression of HER by electrolyte additives: (i) modification of the electrode surface by an additive, leading to increased hydrophobicity and thereby displacing water from the electrode surface, and (ii) electrostatic interactions caused by the presence of charged additive species within the electrochemical double layer (EDL), potentially stabilizing reaction intermediates.^18–20^ Wang et al. investigated the reduction of an imidazolium-based IL on a Cu electrode, revealing the formation of an [EMIM]^+^ based layer that acted as an active catalytic site for the CO_2_RR on the electrode surface. This layer effectively suppressed the HER by inhibiting proton adsorption.^21^ Alternatively, Lim et al. highlighted the electrostatic effect at the metal/IL interface as a key factor in facilitating CO_2_RR while suppressing HER. Significant polarization of the metal’s electron density toward the key *COO^–^ intermediate in the IL-based electrolyte system occurs, driven by the charge effect of the [EMIM]^+^ cation on the metal surface.^22^ This interaction generates a strong local electric field at the EDL interface, stabilizing the *COO^–^ intermediate through field–dipole interactions. The effect of electrolyte additives has been predominantly demonstrated in the simple batch cells, with model catalysts and at very low current densities.^7,14,21–23^ The poor mass transport of CO_2_ through electrolytes significantly limits the current density of the CO_2_RR in the batch cell setup. Therefore, demonstrating the scalability of the additive effect in flow cell configurations, which are more suitable for industrial applications, is essential. A rare example by Han et al. reported the use of ethylenediamine tetramethylenephosphonic acid (EDTMPA) as an electrolyte additive to achieve high CO_2_-to-CH_4_ conversion at elevated current densities of up to 300 mA/cm^2^ in flow cell configuration.^24^ Nevertheless, further insight concerning the influence of electrolyte additives in nonmodel systems and practically relevant gas diffusion electrode (GDE) configurations is required for understanding the underlying mechanisms also at higher current densities.

In this study, we investigate the potential of sorbitol as an electrolyte additive for modifying the local environment near the electrode–electrolyte interface for the CO_2_RR in the GDE flow cell electrolyzer configuration. Sorbitol is a cheap, sustainable, and biobased molecule, that enables the effective suppression of HER, previously utilized for galvanic metal deposition, but not for CO_2_RR.^25,26^ Molecular dynamics (MD) simulations reveal that adding sorbitol to the electrolyte affects the K^+^ and HCO_3_^–^ ions’ presence at the electrode–electrolyte interface. Our mechanistic studies suggest that sorbitol plays a crucial role in controlling the local ion concentrations (of cations and anions) and the interactions between cations and CO_2_/H_2_O molecules at the GDE electrode surface. Consequently, CO production is enhanced, increasing the Faradaic efficiency (FE) from 79% to 90%, while HER is suppressed, reducing the FE from 15% to 5% at current densities up to approximately 100 mA·cm^–2^ in the GDE flow cell electrolyzer. The effect of sorbitol as a nonionic electrolyte additive is distinct from the commonly reported ionic liquid or surfactant-based additives, which primarily offer hydrophobic shielding of the catalyst. Instead, sorbitol introduces an alternative indirect mechanism for optimizing the CO_2_RR pathway and suppressing HER by modulating competitive molecular interactions among the various active species near the electrode surface during both the CO_2_RR and HER processes. This can serve as a starting point for the development of a novel class of additives for the CO_2_RR. Furthermore, the usability of sorbitol as an additive was demonstrated at higher current densities and in practically relevant GDE flow cell configurations.

## 2 Results and Discussion

The suppression of HER in electrochemical reactions not only is a challenge for the CO_2_RR but also impacts the efficiency of other processes such as galvanic metal deposition processes from aqueous electrolytes. In these deposition processes, HER can deteriorate the quality of the deposited metal layers, reducing the overall process efficiency. Sorbitol as an additive has been reported to positively affect those metal depositions by partially suppressing the HER.^25,26^ This highlights its potential as an additive for enhancing the CO_2_RR as well. In this study, silver (Ag) nanoparticles were selected as an ideal model system and used as the CO_2_RR catalyst, by supporting them on a carbon-based GDE. A flow cell, consisting of three compartments; gas diffusion chamber, catholyte chamber, and anolyte chamber (shown in Figure S1), was employed for the CO_2_RR experiments. Potentials were measured versus an Ag/AgCl leakless electrode. The Ag/GDE (as shown in Figure S2) system produced exclusively CO, H_2_, HCOOH and a tiny amount of CH_4_, consistent with previous reports.^27,28^ To evaluate the influence of the additive, sorbitol was directly added to the aqueous catholyte (0.5 M KHCO_3_) while all other experimental parameters remained constant.

FE and partial current densities (PCD) were employed to compare the CO_2_RR activity and selectivity with and without sorbitol. At first, the effect of increasing the sorbitol concentration from 0 to 500 mM was evaluated at a constant current density of 29.4 mA·cm^–2^ (Figure 1a). The introduction of sorbitol exhibits a clear beneficial effect, inhibiting HER across increased sorbitol concentrations while enhancing the FE for CO. The maximum FE for CO of 89.6% was achieved at 100 mM sorbitol concentration, with a concurrent HER of only 4%, compared to 79.1% FE for CO and 10.9% HER, when no sorbitol additive was used. The FE for CO do not significantly change after increasing the sorbitol concentration beyond 100 mM. The FE for methane is minimal and remains unaffected by the increase in sorbitol concentration. In the next step, the effect of sorbitol at various current densities ranging from 9.8 mA·cm^–2^ to 98 mA·cm^–2^ was investigated. In Figure 1b, a direct comparison of FE between the no-additive case and the presence of 100 mM sorbitol is illustrated for various current densities in the flow cell. Improvement in FE for CO and suppression of HER were observed across all current densities, with a minor increase in the formic acid FE with the presence of sorbitol. Increasing the sorbitol concentration from 0 to 100 mM at 98 mA·cm^–2^ shows a significant enhancement of FE for CO from 75.6% to 79.0% and remarkable HER suppression from 15.1% to 5.4%. This underscores the positive impact of sorbitol on promoting CO_2_RR and suppressing HER. This fact is further underlined by Figure 1c, where the H_2_/CO ratio (%) is illustrated at different current densities, reaching its lowest point at 29.4 mA·cm^–2^ with the presence of sorbitol. The H_2_/CO ratio remains significantly lower in the presence of sorbitol than in the no-additive case across all current densities. The largest difference occurs at a current density of 98 mA·cm^–2^, which is critical for enhancing the CO_2_RR while minimizing HER at higher current densities. Furthermore, in Figure 1d, the partial current densities (PCD) of CO and H_2_ were compared with and without sorbitol. Across the total current density range of 9.8 mA·cm^–2^ to 98 mA·cm^–2^ PCD of CO exhibited an increase in the presence of Sorbitol, with the concurrent decrease of PCD for H_2_.

**Figure 1:**
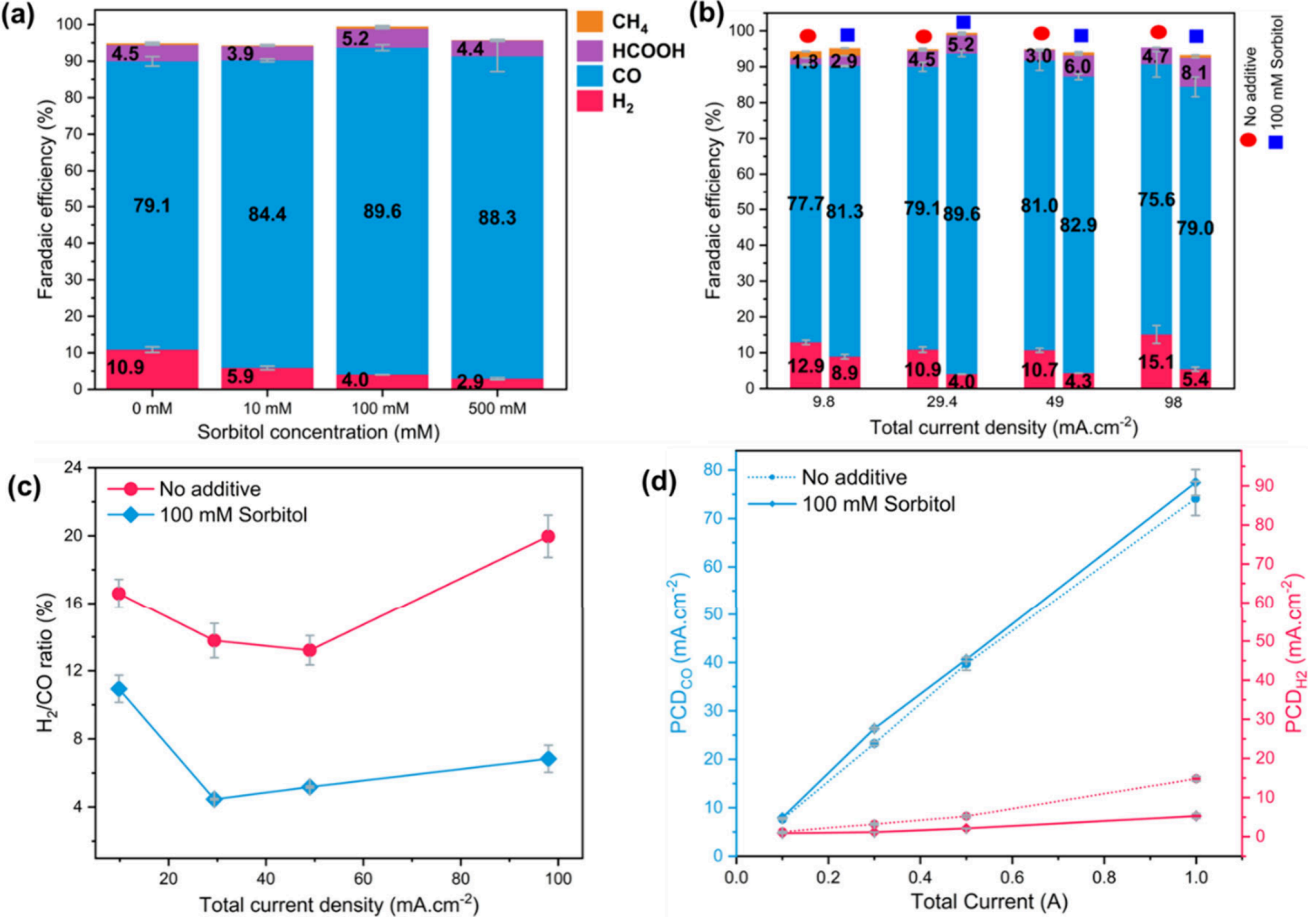
Faradaic efficiency of CO_2_RR products. (a) Sorbitol concentration variation from 0 mM to 500 mM at 29.4 mA·cm^–2^ current density. (b) Comparison of Faradaic efficiencies between no additive (left columns) and 100 mM sorbitol additive (right columns) at different current densities. (c) H_2_/CO ratio (%) without additives and with 100 mM sorbitol at different current densities. (d) Partial current density (PCD) of CO and H_2_ without additives and with 100 mM sorbitol. CO_2_RR was performed in a flow cell with Ag loaded GDE and 0.5 M KHCO_3_ as initial catholyte (to which sorbitol was added directly if required).

Chronopotentiometry, as depicted in Figure 2a, shows that the addition of sorbitol up to concentrations of 100 mM does not lead to a significant potential shift. This is consistent for all investigated current densities, as illustrated in Figure 2b and Figure 2d. The chrono-potentials at the same current density are equal to both the no-additive case and the 100 mM sorbitol case (approximately −1.9 V). A significant potential shift to approximately −2.05 V is, however, visible for the concentration of 500 mM sorbitol (Figure 2a). As demonstrated by electrochemical impedance spectroscopy (EIS) data, as shown in Figure S3, this shift corresponds to a significant increase of charge transfer resistance from 398.7 to 626.8 Ω along with a minor increase in the solution resistance from around 1.2 to 1.44 Ω. To elucidate long-term stability, CO_2_RR was conducted for 10 h at 29.4 mA·cm^–2^ with 100 mM sorbitol, as depicted in Figure 2c. Following continuous CO_2_RR operation in the flow cell, the long-term CO FE only slightly decreased from 89% to 85% over the 10-h duration. In contrast, under identical conditions without the addition of sorbitol, the CO FE decreased significantly from 80.8% to 54.4% after 10 h of chronoamperometry at 29.4 mA·cm^–2^. Additionally, notably, a sharp increase in cell potential was observed after 6.5 h in the absence of the additive, suggesting a significant change in electrode behavior. These observations underscore the critical role of sorbitol in suppressing electrolyte flooding within the GDE and sustaining the CO_2_RR selectivity during extended operation. The substantial reduction in CO FE and abrupt rise in cell potential in the absence of sorbitol are consistent with progressive electrolyte intrusion, which limits transport of CO_2_ to the catalyst layer and facilitates the competing hydrogen evolution reaction (HER). The elevated HER activity can further accelerate GDE flooding, exacerbating the loss of CO_2_RR selectivity as previously reported in the literature.^29^ These findings highlight the good stability of the Ag-GDE catalyst during extended CO_2_ electrochemical reduction and further underscore that the beneficial effect of sorbitol can be utilized over long reaction times.

**Figure 2:**
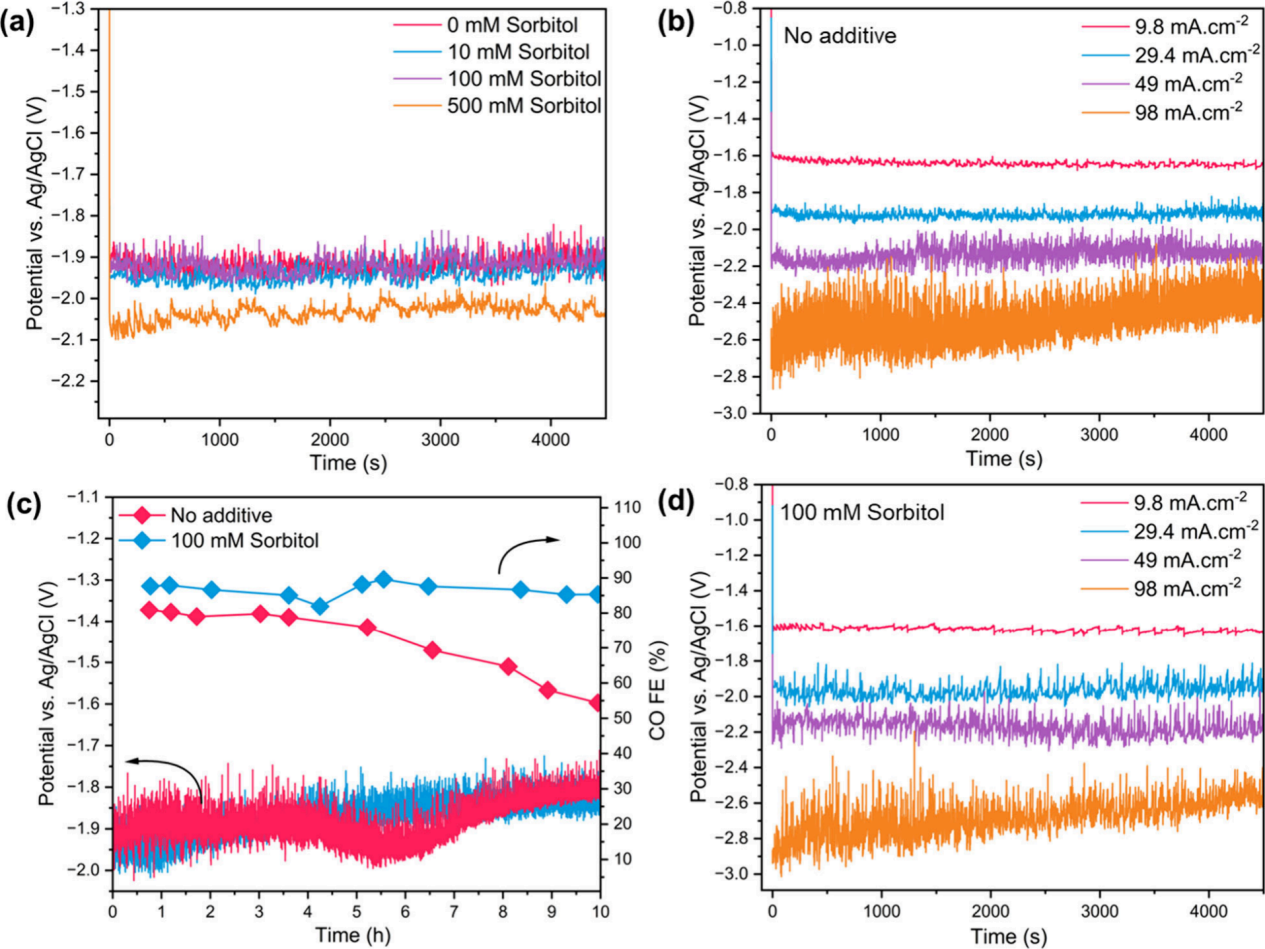
Chronopotentiometry of (a) Ag at 29.4 mA·cm^–2^ current density with different sorbitol concentrations, (b) with no additive at different current densities. (c) Stability of Ag-GDE with 100 mM sorbitol for 10 h in the flow cell, and (d) Ag with 100 mM concentration of sorbitol at different current densities. CO_2_RR was performed in flow cell with Ag loaded GDE and 0.5 M KHCO_3_ as initial catholyte (to which sorbitol was added directly if required).

### Control Experiments

As sorbitol is a carbonaceous additive, it must be ensured that the increased FE for CO and formic acid are indeed caused by an enhancement of the CO_2_RR and not from a hypothetical degradation of sorbitol itself. Therefore, a control experiment was conducted to assess the effect of sorbitol on the electrochemical reaction in the absence of CO_2_. Instead, argon (Ar) was flowed continuously to the gas compartment of the electrolyzer, while the concentration of sorbitol in the electrolyte was kept at 100 mM. The catalyst performance was tested by chronopotentiometry for 4500 s at a current density of 29.4 mA·cm^–2^. As shown in Figure S3a, the CO_2_-saturated electrolyte exhibits an onset potential of approximately −1.2 V vs Ag/AgCl. In contrast, the Ar-saturated electrolyte shows an initial onset near −0.3 V vs Ag/AgCl, can be attributed to HER facilitated by proton donation from bicarbonate ions, followed by a current plateau and a second pronounced increase at around −1.55 V vs Ag/AgCl, corresponding to the onset of direct water reduction.^30^ The suppression of this early HER feature under CO_2_ and the shift to more negative onset are consistent with electrolyte-driven interfacial reorganization and competition by CO_2_ reduction intermediates.^31,32^ Cyclic voltammetry results in Figure S3b showed little variation, when changing sorbitol concentrations up to 100 mM. However, at 500 mM sorbitol, a lower current was observed at –1.4 V vs. Ag/AgCl, which can be attributed to the higher charge transfer resistance, as confirmed by the impedance spectroscopy data in Figure S3d. Furthermore, in the presence of Ar in the gas feed (and sorbitol in the liquid feed), hydrogen was detected by gas chromatography (GC) as the sole gaseous product and additionally no formic acid was found in the liquid phase by high-performance liquid chromatography (HPLC) as shown in chromatograms in Figure S5a and Figure S5b, respectively. In contrast, when CO_2_ was introduced into the gas compartment with sorbitol present in the electrolyte, both CO and formic acid were detected, as shown in Figure S5c and Figure S5d, respectively. In addition, the sorbitol concentration was measured before and after CO_2_RR, which was performed under chrono potentiometric conditions at a current density of 29.4 mA·cm^–2^ for 1 h, using 100 mM sorbitol as an additive in the electrolyte. No change in the sorbitol concentration was observed before and after the CO_2_RR, as shown in the HPLC chromatogram in Figure S6. This confirms that the increase in CO_2_RR products can be truly attributed to CO_2_ electroreduction but not to degradation of sorbitol within the flow cell.

### Material Characterization

The morphology and chemical composition of the GDE/catalyst assembly before and after CO_2_RR was analyzed using scanning electron microscopy (SEM), energy dispersive X-ray spectroscopy (EDX), and X-ray photoelectron spectroscopy (XPS). SEM images with low magnification in Figure S8 confirm the uniformity of the Ag nanoparticle layer on the GDE substrate. Figure 3 presents a high-magnification SEM image offering detailed morphological insights into the deposited catalyst particles. It reveals that individual silver nanoparticles, approximately 100 nm in size, are agglomerated to form structures resembling 1 μm-sized spheres. EDX analysis, as illustrated in Figure 3, revealed the presence of carbon from the substrate and chlorine from the Sustainion ionomer binder, alongside silver. The SEM images and elemental mapping for the post-CO_2_RR materials without sorbitol and with a 100 mM sorbitol additive are presented in Figures S9 and S10, respectively. The catalyst exhibited a good stability during electrolysis, with no discernible changes in morphology was observed. Independent of sorbitol addition, elemental analysis via EDX revealed the presence of potassium, as shown in Figures S9 and S10, which can be attributed to the salt precipitation of KHCO_3_ on the GDE, after electrochemical tests. Additionally, in the case of sorbitol, a minor increase in oxygen content was noted, potentially stemming from residual traces of sorbitol remaining on the GDE post-CO_2_RR. XPS analysis (Figure S11) detected silver, chlorine from the Sustainion ionomer, and carbon, fluorine, and oxygen from the GDE substrate. The stability of the catalyst post-CO_2_RR is evident as no shift in the silver peak was observed (Figure S11b). The C 1s spectrum (Figure S11c) showed increased C–O and C=C/C–H components after the CO_2_RR, with further enhancement in the presence of sorbitol. The O 1s spectrum (Figure S11d) broadened post-CO_2_RR due to an increase in the H_2_O component, with additional C–O component enhancement in the sorbitol-treated sample.

**Figure 3:**
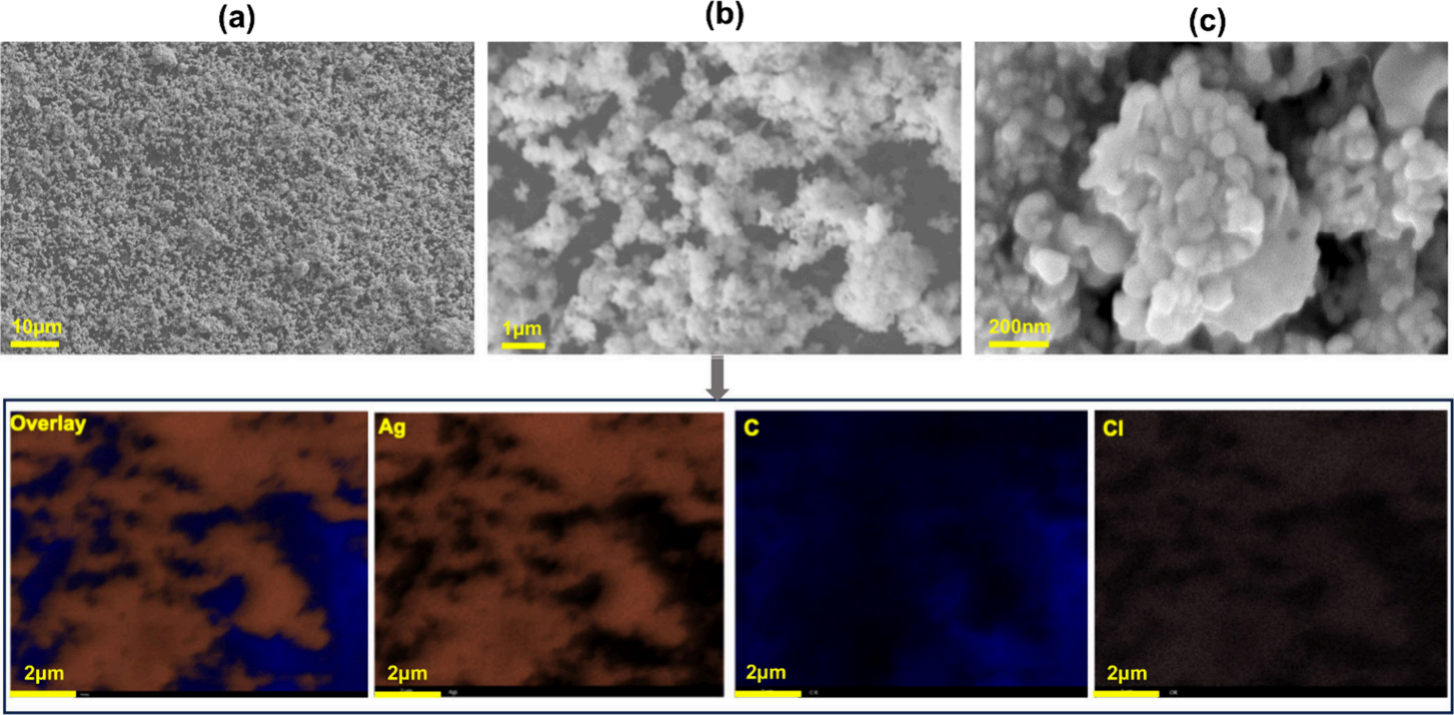
SEM images of the Ag-GDE before the CO_2_ reduction reaction (CO_2_RR) at different magnifications: (a) 1200 fold, (b) 10000 fold, and (c) 61000 fold. Corresponding EDX elemental mapping of image (b) showing the distribution of elements.

While the effectiveness and beneficial nature of sorbitol as an electrolyte additive have been clearly demonstrated in the electrochemical tests, the molecular mechanism resulting in those improvements is yet to be discussed. Other studies demonstrated that additives could tune the activity and selectivity of the CO_2_RR by modifying the local environment at the electrode/electrolyte interface.^6,14,33^ Even though most of these studies were performed at a very low current density and not with realistic GDE setups, different interaction mechanisms have been identified. For ionic liquid additives, it was found that their anions can influence local pH, which then affects CO_2_RR activity by altering the local CO_2_ concentration.^14^ In many cases, the hydrophobic nature of the additive is utilized to reduce the local concentration of water, resulting in decreased HER.^34^

### Molecular Dynamics Simulations

To gain a deeper understanding of how sorbitol influences the local environment at the interface between the Ag electrode and the aqueous electrolyte, molecular dynamics (MD) simulations were performed. The resulting species concentration profiles near the electrode, obtained for various sorbitol concentrations, are presented in Figure 4. The corresponding number of molecules at the interface (within 6 Å to the surface) is summarized in Table S6. Increasing the sorbitol content in the solution results in the accumulation of sorbitol molecules in the interfacial region. As shown in Figure 4(a), two distinct layers of sorbitol emerge at the interface: one layer consists of molecules directly adsorbed onto the electrode surface, while the second layer is positioned right behind the first layer, further away from the surface. The CO_2_ concentration, shown in Figure 4(b), remains largely unaffected with added sorbitol; however, a significant impact is observed for the bicarbonate concentration at the electrode–electrolyte interface, depicted in Figure 4(c). With increasing sorbitol concentration, the amount of bicarbonate decreases. This reduction can directly influence the HER. Koper et al. have previously studied the role of bicarbonate buffer on HER in mildly alkaline conditions, showing that a decrease in bicarbonate concentration is detrimental to HER by disrupting the bicarbonate-mediated HER pathway in such environments.^30^ The reduced interfacial concentration of bicarbonate therefore indirectly benefits the CO_2_RR by suppressing the competitive HER.

**Figure 4:**
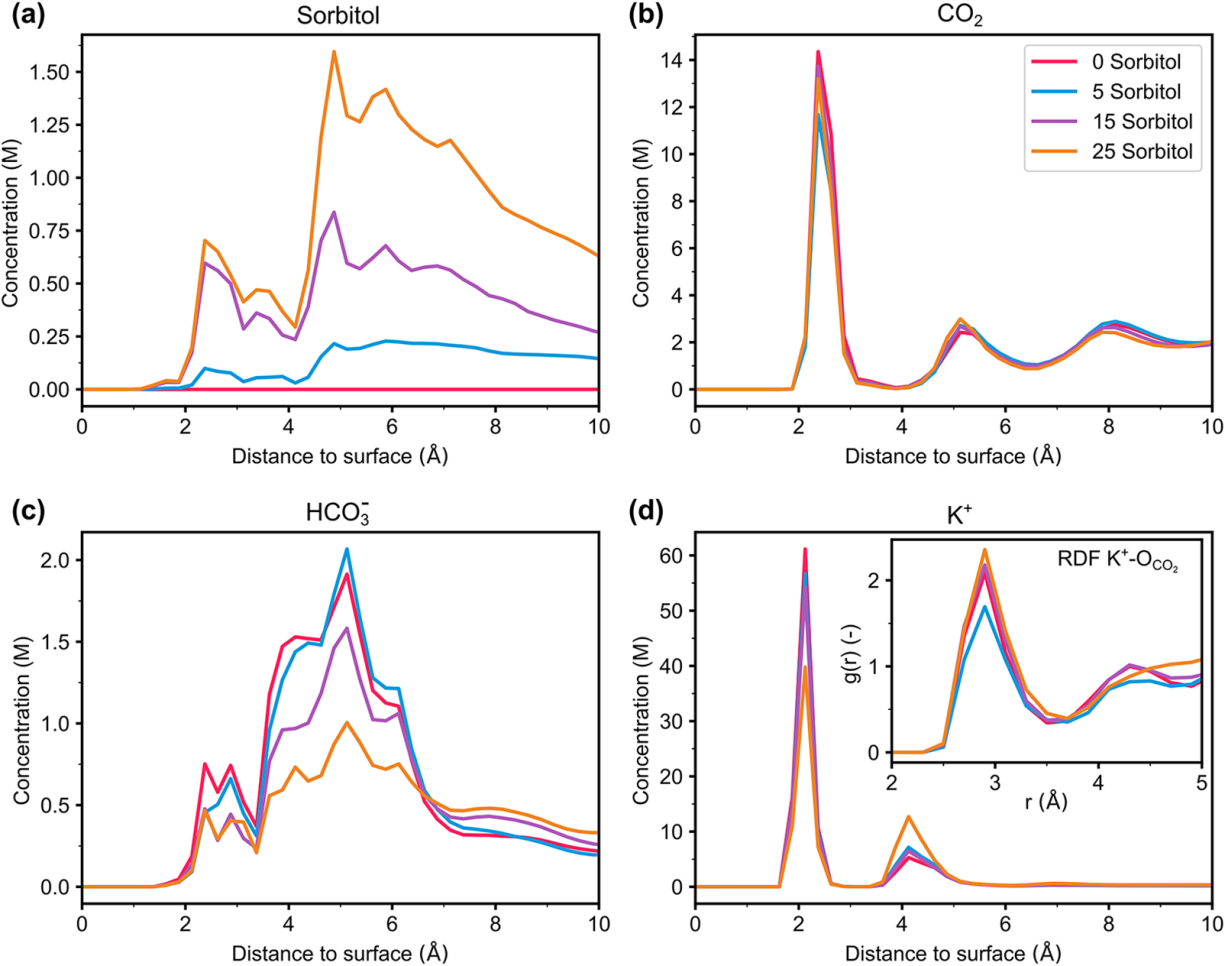
Concentration profiles in the vicinity of the Ag surface of (a) Sorbitol, (b) CO_2_, (c) HCO_3_^–^ and (d) K^+^ ions for different amounts of sorbitol molecules added to the aqueous solution. The inset in (d) shows the RDF between the K^+^ ions located within the first 6 Å to the surface and the oxygen of the CO_2_ molecules.

As shown in Figure 4(d), the K^+^ concentration exhibits two distinct peaks at the interface. Increasing the sorbitol concentration yields a monotonic decrease of K^+^ ions in the first peak that leads to a shift of the ions to the second peak, keeping the total number of cations that are located within the first 6 Å to the surface almost constant. This behavior shows that sorbitol molecules preferentially adsorb on the surface, effectively removing K^+^ ions from the surface. The calculated coordination of K^+^ ions with CO_2_ molecules can be seen in the radial distribution functions as the inset of Figure 4(d). For the two highest sorbitol concentrations studied, the interaction of K^+^ with CO_2_ in the first coordination shell is increased compared with the no-additive case. For a CO_2_ bulk concentration of 2 M that was obtained in the simulations (see supplementary Figure S12), the calculated number of CO_2_ molecules in the first coordination shell of K^+^ is 0.186, 0.183, 0.202, and 0.242 for increasing sorbitol concentration. In contrast, the coordination of K^+^ with water molecules was only slightly affected by sorbitol at the interface (as seen in Figure S12a and S12b). This limited impact arises primarily because water–water interactions are inherently stronger than those between water and sorbitol molecules. This distinction is clearly reflected in the RDFs presented in Figure S13. The densely packed nature of water molecules near interfaces results in an environment that is less accommodating to the larger sorbitol molecules. Consequently, sorbitol tends to position itself slightly farther from the surface, around the outer Helmholtz plane. This spatial arrangement underscores a key point: the suppression of hydrogen evolution reaction (HER) at these interfaces is not primarily due to alterations in water organization but rather results from the exclusion of bicarbonate ions. Thus, while the sorbitol’s presence affects certain molecular interactions, its influence on K^+^ coordination with water is minimal, highlighting that the critical factor for HER suppression is the reduced accessibility of bicarbonate ions to the interface. This finding emphasizes the role of ionic composition over mere changes in the structural arrangement of water molecules on charged surfaces. The minimal impact of sorbitol on the coordination dynamics between K^+^ and water molecules at the interface further points toward the significant role of alkali metal ions in influencing CO_2_RR activity as has been extensively explored in previous studies.^35–37^ It was demonstrated that cations, such as K^+^, stabilize negatively charged reaction intermediates, such as *CO_2_^–^ and *OCCO^–^.^37^ Therefore, the increased K^+^-CO_2_ coordination due to the presence of sorbitol might further benefit the CO_2_RR by enhancing the stabilization of these negatively charged intermediates. This effect can be attributed to the stronger electrostatic interactions between K^+^ ions and the reaction intermediates, facilitated by the presence of sorbitol. This means that besides the minimization of interfacial bicarbonate, the increased interaction between K^+^ and CO_2_ can be a second phenomenon to explain the beneficial nature of sorbitol. The ability of sorbitol to influence the local ionic environment highlights its potential as an additive in electrocatalytic processes. These findings underscore the importance of tuning the electrolyte composition to optimize the CO_2_ reduction reaction pathways and overall catalytic performance. In addition to these findings, we have extended our study by conducting simulations with partially deprotonated sorbitol molecules, as illustrated in Figure S14. These simulations further elucidate the behavior and interactions of sorbitol in high pH environments, where the OH-groups of sorbitol are partially deprotonated. Trends of density profiles and RDFs show no significant deviation from those involving fully protonated sorbitol molecules, as shown in Figure S15 and Figure S16. Thus, the overall conclusions regarding the suppression of the hydrogen evolution reaction (HER) and the enhancement of CO_2_ coordination with increased sorbitol remain unchanged, reinforcing the robustness of our results across different pH conditions.

To further elucidate the mechanistic influence of sorbitol on CO_2_RR, Tafel analyses were conducted for both the CO_2_RR and HER. As depicted in Figures 5a and 5b, the Tafel slope for the HER increased from 229 to 273 mV/dec, while for the CO production, it decreased from 132 to 103 mV/dec with the addition of 100 mM sorbitol, respectively. This rather minor change in the Tafel slope for the CO_2_RR suggests that sorbitol does not drastically alter the fundamental reaction mechanism or rate-determining steps for the CO_2_ reduction. Introducing 100 mM sorbitol into the electrolyte notably suppresses the partial current density for H_2_ evolution (Figure S4a) while concurrently enhancing the partial current density for CO production (Figure S4b) across the investigated potential range. These results indicate that sorbitol selectively suppresses the HER and enhances the CO_2_ to CO conversion.

**Figure 5:**
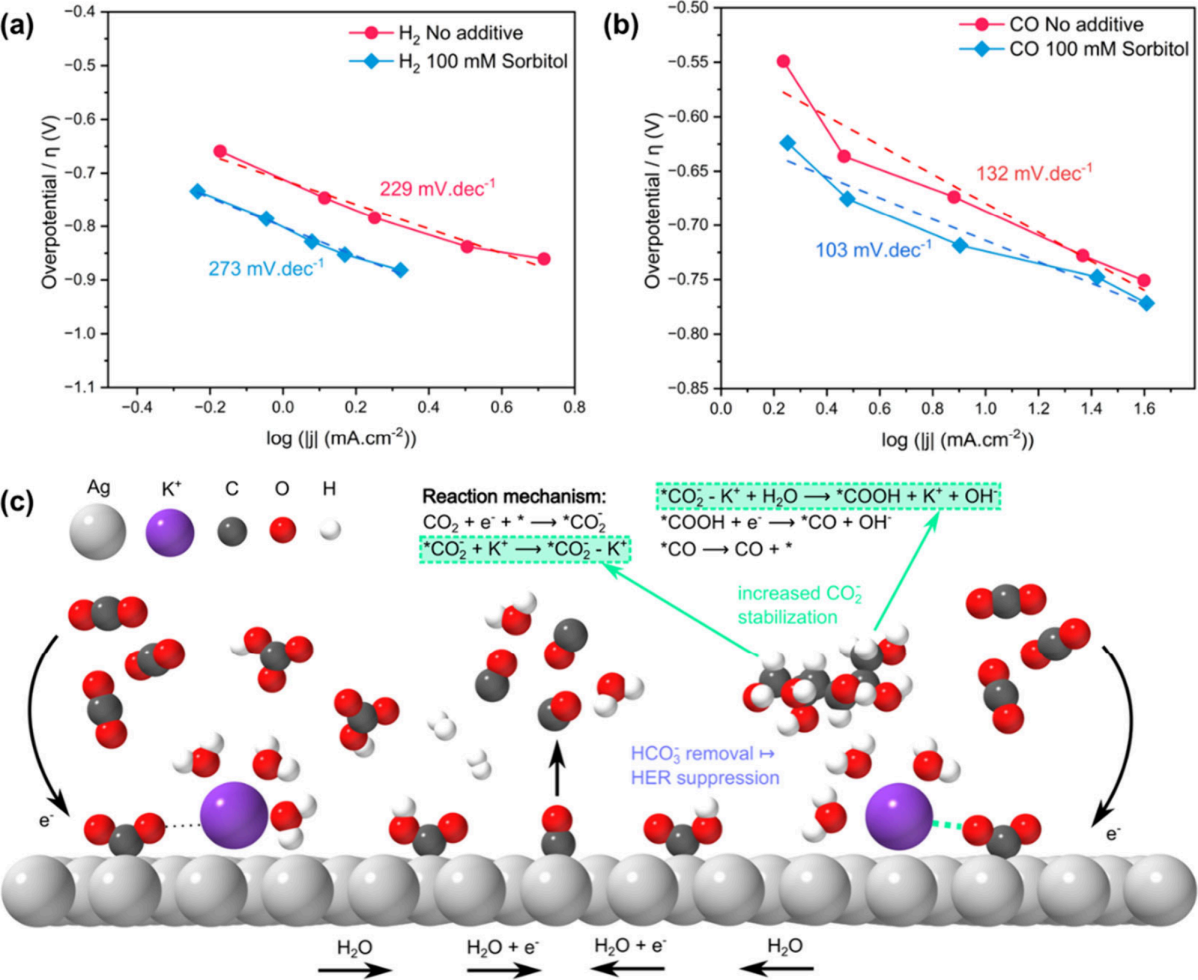
Tafel plots for (a) H_2_ production and (b) CO production over Ag GDE at 0 mM sorbitol concentration and 100 mM sorbitol additive concentration with the CO_2_ flow. (c) Schematic representation of the interaction of potassium ions with the negatively charged CO_2_ intermediates together with a proposed reaction mechanism of electrochemical reduction of CO_2_ to CO.

The two potential molecular mechanisms, explaining the beneficial nature of sorbitol, are illustrated in Figure 5c. The increased K^+^-CO_2_ coordination and decreased bicarbonate ions at the electrode/electrolyte interface in the presence of sorbitol are shown. These phenomena differ from the mechanisms attributed to ILs or surfactant-based additives. Sorbitol directs a complex cascade of ion interactions and assemblies, without being directly involved in the reaction mechanism itself. These results demonstrate that additives, including molecular compounds beyond surfactants or ionic liquids, can be an effective pathway to enhance the performance of the CO_2_RR, opening new possibilities for reaction optimization.

## 3 Conclusion

This study showed that sorbitol, as a cheap and sustainable additive, improves the electrochemical CO_2_ reduction by substantially increasing the CO Faradaic Efficiency (FE) to 89.6% while concurrently suppressing the HER to 4%. This positive impact was demonstrated in a flow cell electrolyzer with a silver nanoparticle catalyst/GDE assembly, at current densities of up to 98 mA·cm^–2^ and reaction times up to 10 h. Unlike prior studies that primarily employed additives in batch cells at low current densities, this work underscores the applicability of sorbitol for CO_2_RR at practically relevant reaction systems and current densities. Our findings shed light on the molecular effects of sorbitol influencing product selectivity and activity in CO_2_ reduction reactions. Molecular dynamics (MD) simulations revealed that sorbitol significantly modulates the local electrode/electrolyte interfacial environment. The addition of sorbitol reduced the interfacial bicarbonate ion concentration, disrupting HER pathways while enhancing the coordination between potassium ions and CO_2_. This enhanced K^+^-CO_2_ interaction likely stabilizes reaction intermediates, such as *CO_2_^–^, facilitating the formation of CO and additionally improving the CO_2_RR efficiency additionally. Furthermore, the suppression of HER by sorbitol stems from its indirect impact on interfacial ion dynamics rather than the hydrophobic shielding commonly attributed to ionic liquid or surfactant-based additives. This distinct mechanism broadens the scope of molecular additives beyond conventional ionic liquids and surfactants, paving the way for the development of new additive strategies for the CO_2_RR optimization.

### Experimental Section

#### Materials and Instrumentation

Silver nano powder (<100 nm particle size, 99.5% trace metals basis); sorbitol (99%), KHCO_3_ (99.5%) and KOH (99.9%) were purchased from Sigma-Aldrich and used as received. PTFE-treated carbon papers with a microporous layer (Freudenberg H23C6) were obtained from Fuel Cell Store and afterward cut into desired dimensions (3 × 3.4 cm^2^) using a razor blade. The entire micro flow cell assembly, including a leakless Ag/AgCl micro reference electrode and an IrO_2_-coated titanium plate for the counter electrode, was sourced from Electrocell (microflow cell). Sustainion X37-50 grade RT anion exchange membranes (AEMs) and Sustainion XA-9 Alkaline Ionomer (5 wt %, in a mixture of lower aliphatic alcohols and water) were purchased from Dioxide Materials. AEMs were transformed into the hydroxide form by immersion in 1 M KOH for more than 24 h. Electrolyte and electrode preparations followed suit accordingly.

#### Electrode Preparation

A silver (Ag) catalyst ink was prepared by dissolving 10 mg of catalyst and 100 μL of Sustainion XA-9 Alkaline Ionomer in 1 mL of isopropyl alcohol, followed by ultrasonication for 90 min. Subsequently, the catalyst ink was applied using manual airbrushing onto a 10.2 cm^2^ hydrophobic carbon paper with a microporous layer (Freudenberg paper H_2_3C6), with an argon pressure of 1.5 bar in the airbrush, see supplementary Figure S2. To expedite solvent evaporation during manual airbrushing, the hot plate beneath the gas diffusion electrodes (GDEs) was set to 150 °C. The carbon paper was weighed before and after catalyst deposition, resulting in a catalyst mass loading of approximately 0.6 mg·cm^–2^ after drying.

#### Electrochemical Experiments in the Flow Cell

All electrochemical experiments were conducted within a microflow cell purchased from Electrocell, as illustrated in Figure S1, with electrode areas of 10.2 cm^2^. The cell is composed of three compartments: one for the anolyte, another for the catholyte, and a third for the gas compartment. All of the electrochemical experiments were conducted at room temperature and pressure using an Autolab PGSTAT302N potentiostat in a three-electrode flow cell setup. An IrO_2_-coated Ti current collector plate served as the anode, while a leakless Ag/AgCl micro reference electrode was used as the reference electrode. Freshly prepared 0.5 M KHCO_3_ and 0.5 M KOH solutions were used as the catholyte and anolyte, respectively, and were circulated at a flow rate of 45 mL/min using a peristaltic pump within the flow cell. CO_2_ (or Ar for control experiments) was introduced at a flow rate of 60 sccm. The catalyst GDE, anion exchange membrane (AEM), and anode plate were assembled within the flow cell, and the electrolytes and gas were circulated for 30 min to saturate the system. Electrochemical stability was achieved through 15 cyclic voltammetry (CV) scans ranging from 0 to −1.5 V versus the Ag/AgCl reference electrode. All CV and linear sweep voltammetry (LSV) experiments were performed at a consistent scan rate of 100 mV/s, (see supplementary Figure S3) with potential values fully corrected for internal resistance (IR). Chronopotentiometry was then employed to assess CO_2_RR performance at various current densities over 4500 s (see Figure 2). The ohmic drop was quantified through electrochemical impedance spectroscopy (EIS), where the real component of the Nyquist plot at 1 × 10^3^ Hz provided the value of solution resistance (R_S_). Manual adjustment for 100% IR compensation was carried out during data analysis. The Nyquist plots were fitted utilizing Zview software for comprehensive analysis. EIS measurements were conducted at open circuit potential (OCP) (−0.2 V vs Ag/AgCl) for all additives under both CO_2_ and Ar flows within the same flow cell setup. The resultant Nyquist plots were fitted using Zview software with an equivalent circuit model comprising R_S_ – R_CT_/CPE (see supplementary Figure S3), where R_S_ represents solution resistance, R_CT_ denotes charge transfer resistance, and CPE signifies the constant phase element. Analysis revealed an increase in the solution resistance with increasing concentrations of sorbitol. However, this resistance remained relatively consistent up to a concentration of 100 mM, after which it elevated noticeably for a concentration of 500 mM sorbitol.

#### Product Analysis

The gaseous products’ collection was initiated after 400 s of electrolysis. Gaseous products were collected every 30 min from the outlet of the flow cell into Tedlar gas sampling bags. Samples were then injected into a Shimadzu Nexis GC-2030 gas chromatograph using a gastight syringe. The chromatograph utilized helium as the carrier gas with a total flow rate of 35.4 mL/min under the pressure flow control mode. The pressure was initially set at 226.8 kPa and held for 2.5 min, then increased at 15.2 kPa/min to 390.1 kPa and held for 5.95 min, and finally increased at 11.2 kPa/min to 405.1 kPa, held for 5.42 min. The column used was a ShinCarbon ST Micropacked GC Column (100/120, 1 mm ID, 2 m). The column temperature was ramped from 50 to 270 °C with a column temperature program started at 50 °C and held for 2.5 min then increased at a rate of 10 °C/min to 250 °C, followed by an increase at a rate of 9 °C/min to 270 °C held for 8 min. A barrier ionization discharge (BID) detector was used with a detector temperature of 280 °C and a He discharge flow rate of 50 mL/min.

Cumulative liquid products were analyzed postelectrolysis using a Shimadzu LCMS-2020 liquid chromatograph–mass spectrometer. The analysis was conducted with a Shim-pack SCR-102H ion exchange column (300 mm × 8.0 mm, 7 μm) maintained at 70 °C. Detection was performed with a UV SPD-40 V detector set at a wavelength of 220 nm. The mobile phase flow rate was maintained at 0.8 mL/min.

Electron microscopy was conducted using a Zeiss Sigma EDVP scanning electron microscope (SEM), equipped with an Ametek EDAX Analyzer for energy-dispersive X-ray spectroscopy (EDX) analysis. X-ray photoelectron spectroscopy (XPS) measurements were performed using a Versa Probe III spectrometer (Physical electronics GmbH) at the ELSA cluster at TU Vienna. Monochromated Al Kα (1486.6 eV) was used as radiation, with the beam diameter set to 100 μm and the beam voltage set to 15 kV. The samples were mounted on conductive carbon tape to avoid the charging effect. Survey scans of all samples were recorded at a pass energy of 140 eV and a step size of 0.125 eV. High-resolution core level spectra were recorded at a pass energy of 27 eV and a step size of 0.05 eV. CasaXPS (Fairley, N. CasaXPS, version 2.3.26PR1.0) was used to process the spectra.

#### Details of Molecular Dynamics Simulations

Classical molecular dynamics simulations were performed using Large-scale Atomic/Molecular Massively Parallel Simulator (LAMMPS), version 02/08/2023.^38^ For the simulations, an aqueous solution of KHCO_3_ was confined between two negatively charged Ag surfaces with dimensions of 38.9 Å × 38.9 Å × 12.3 Å, separated by 50 Å as illustrated in Figure S10c. The charging was obtained by applying partial charges to the first two atomic layers of the Ag surface, resulting in a surface charge density of −0.23 C/m^2^. To achieve overall charge neutrality of the system, a surplus of K^+^ ions was added to the solution. For the CO_2_, sorbitol and HCO_3_^–^ molecules, the CHARMM36 force field was used,^39,40^ which has been parametrized using both quantum chemical calculations and experimental data to ensure reliable representation of structural and interaction properties in condensed-phase systems. Water was modeled using the SPC/E parameters,^41^ a well-validated model known to accurately reproduce the thermodynamic and structural properties of liquid water. The Lennard-Jones parameters of the Ag surface and K^+^ ion were obtained from earlier works.^42,43^ The latter is specifically optimized for compatibility with the SPC/E water model and accurately reproduces hydration free energies and the ion–water structure. Cross-correlation terms in the force field were calculated from the Lorentz–Berthelot mixing rules and short-range interactions were cutoff smoothly between 10 and 12 Å. The long-range interactions were calculated with a particle–particle-mesh (PPPM) method.

All simulations were performed with a time step of 0.5 fs in the NVT ensemble at 298 K, keeping the silver surface atoms fixed at their original positions and the O–H bonds and H–O–H angles of water rigid. Data was generated from five independent simulations, each one started from a different initial configuration for high statistical averaging. 5 ns long production runs were used for data collection and averaging after a 2 ns long equilibration time. The initial atom positions for all simulations were generated randomly using PACKMOL.^44^

## Supplementary Material



## Notes

Experimental procedures and schematics, electrochemical data, chromatography data, microscopic images, simulation data and procedures (PDF)
