# Supplementary material for: Using Sorbitol as Electrolyte Additive to Control Interfacial Environments in Electrochemical CO2 Reduction on Silver
Source: ACS Catal. 2025 Sep 16;15(19):16643–52. doi: 10.1021/acscatal.5c04382 (PMC12501930; doi:10.1021/acscatal.5c04382)
Supplement: Supplementary file 1 [file cs5c04382_si_001.pdf]

Using Sorbitol as Electrolyte Additive to Control Interfacial Environments in Electrochemical CO_2 Reduction on Silver

*Anil Kumar Sihag[1,2], Florian Altmann[2], Alper T. Celebi[2], Markus Valtiner[1,2] and Christian M. Pichler[*1,2]*

AUTHOR ADDRESS:

1. Institute of Applied Physics, Vienna University of Technology,1040 Vienna, Austria

2. Center for Electrochemical Surface Technology GmbH, 2700Wr.Neustadt, Austria

*Corresponding author: christian.pic@gmx.at

Supplementary Information

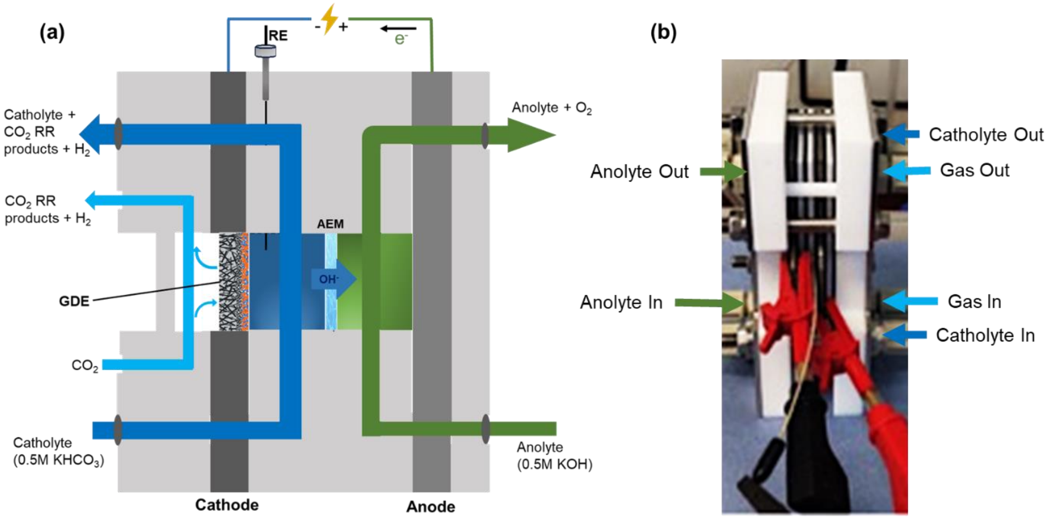

Figure S1. Schematics of CO_2 electrolyzer with three compartments; gaseous compartment, catholyte and anolyte compartment are separated by anion exchange membrane (AEM), Reference electrode (RE) is inserted in the catholyte chamber, and silver (Ag) deposited gas diffusion electrode (GDE).

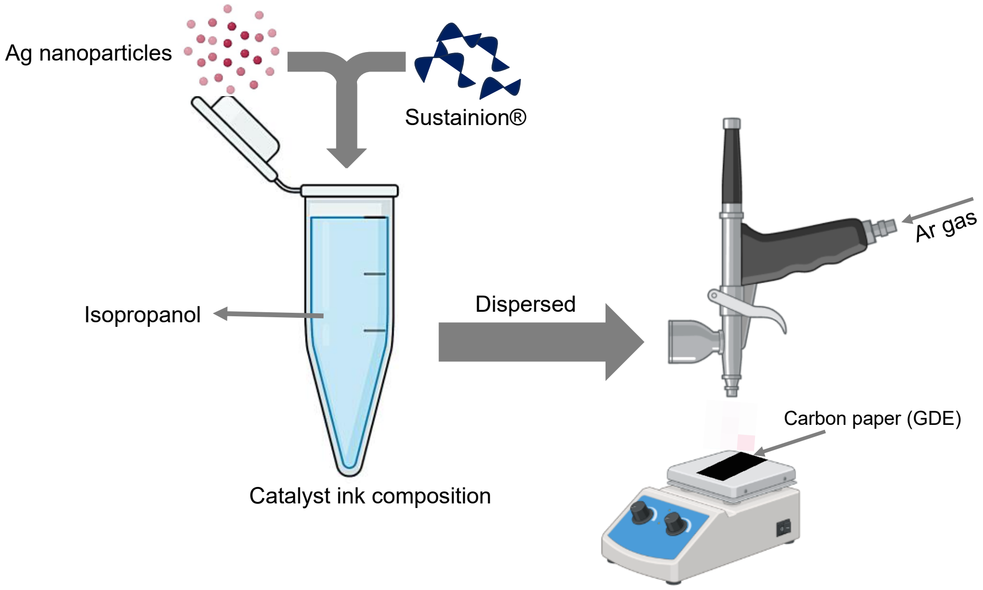

Figure S2. Schematics of the gas diffusion electrode (GDE) process wherein a catalyst ink was prepared by mixing the silver (Ag) nanoparticles and Sustainion ionomer in isopropanol solvent and dispersed in ultra-sonicator for 90 minutes, after dispersed the catalyst was deposited on GDE by airbrushing.

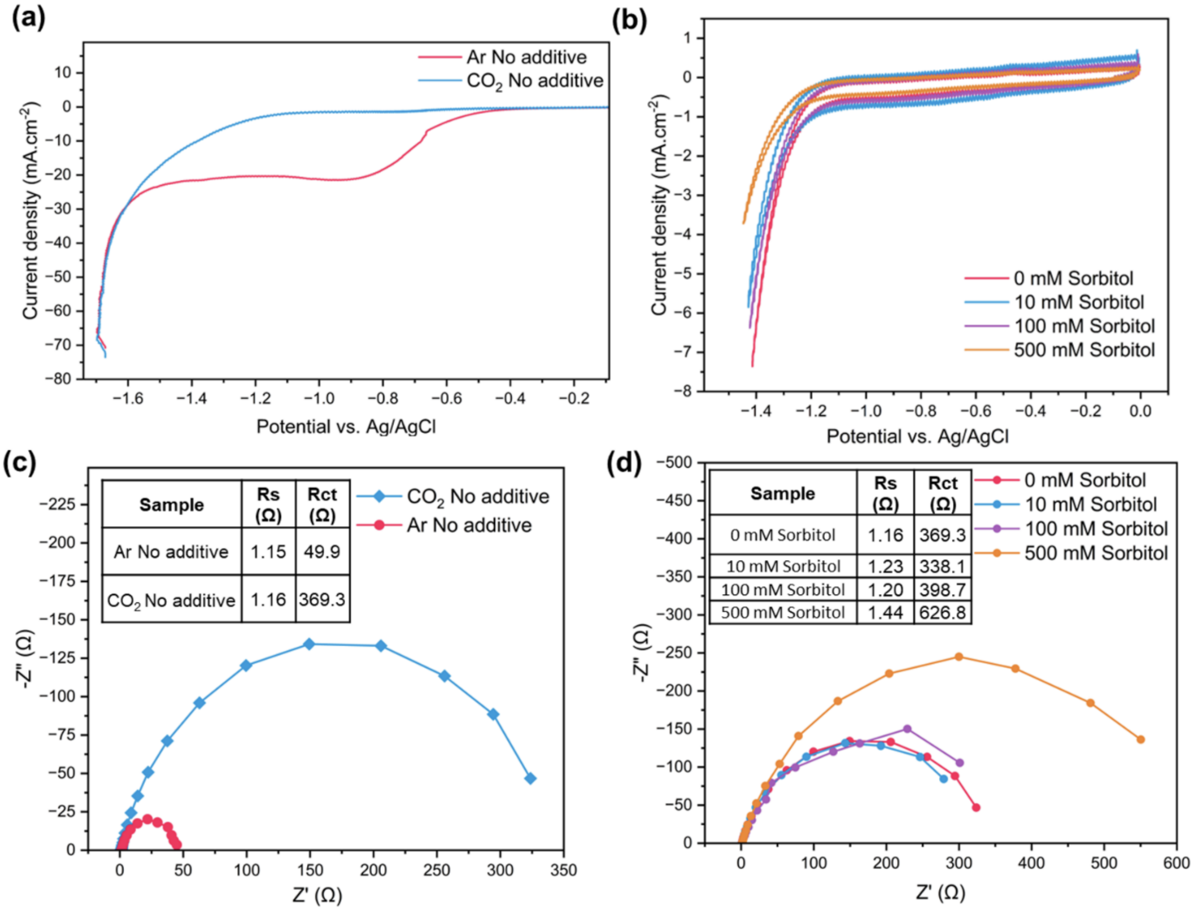

Figure S3. CO_2RR (a) Linear scan voltammetry (LSV) with Ar and CO_2 at 0mM sorbitol concentration, (b) Cyclic voltammetry (CV) with different concentrations of sorbitol with 100% iR compensation applied manually. (c) EIS with Ar and CO_2 at 0mM sorbitol concentration, and (d) EIS at different concentrations of sorbitol.

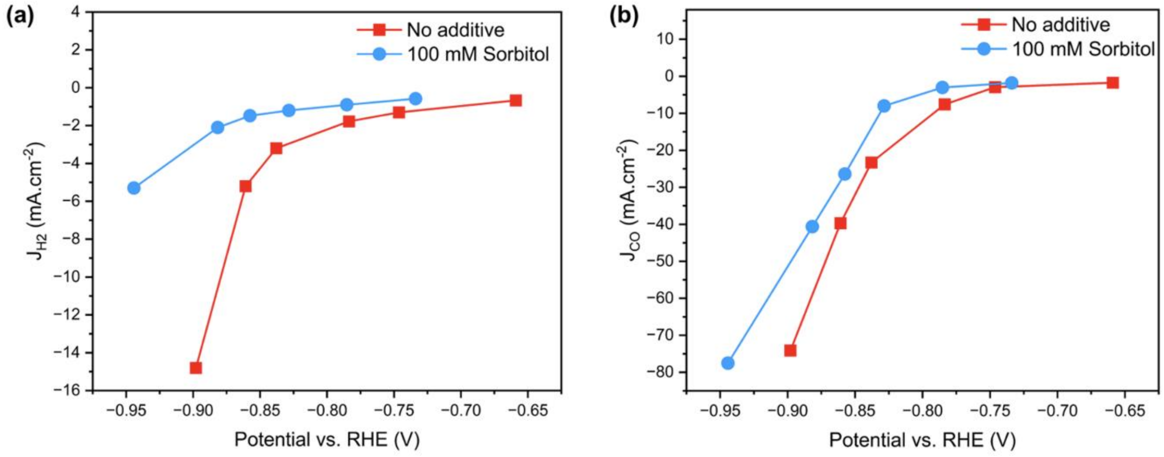

Figure S4. Comparison of Partial current density for CO_2 to H_2 (a) and CO (b) conversion for without additive and with 100 mM sorbitol.

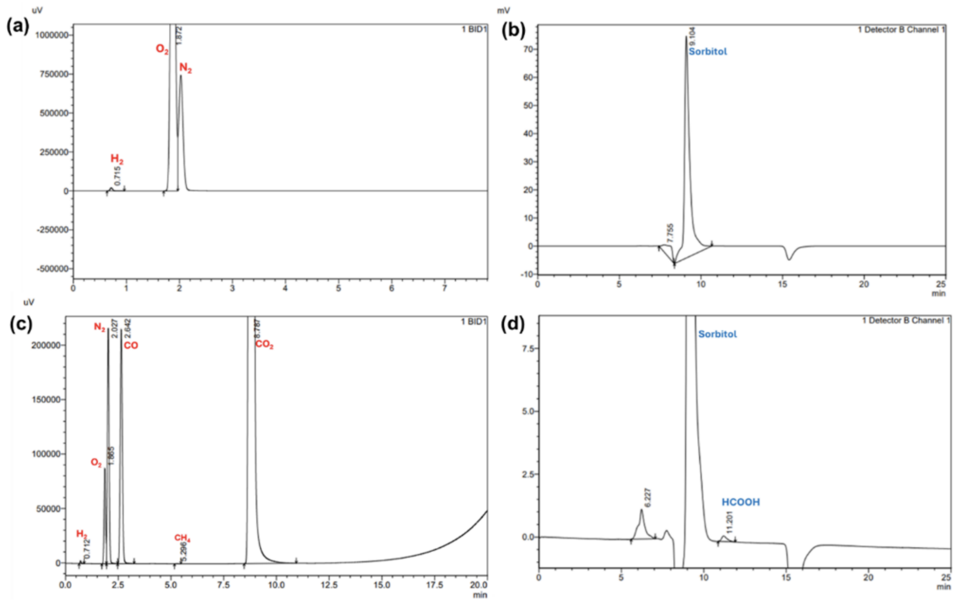

Figure S5. CO_2RR products (a) GC chromatogram, (b) HPLC chromatogram with Ar circulation, (c) GC chromatogram, and (d) HPLC chromatogram with CO_2 circulation at 29.4 $mA.cm^{-2}$ current density chrono with 100 mM Sorbitol.

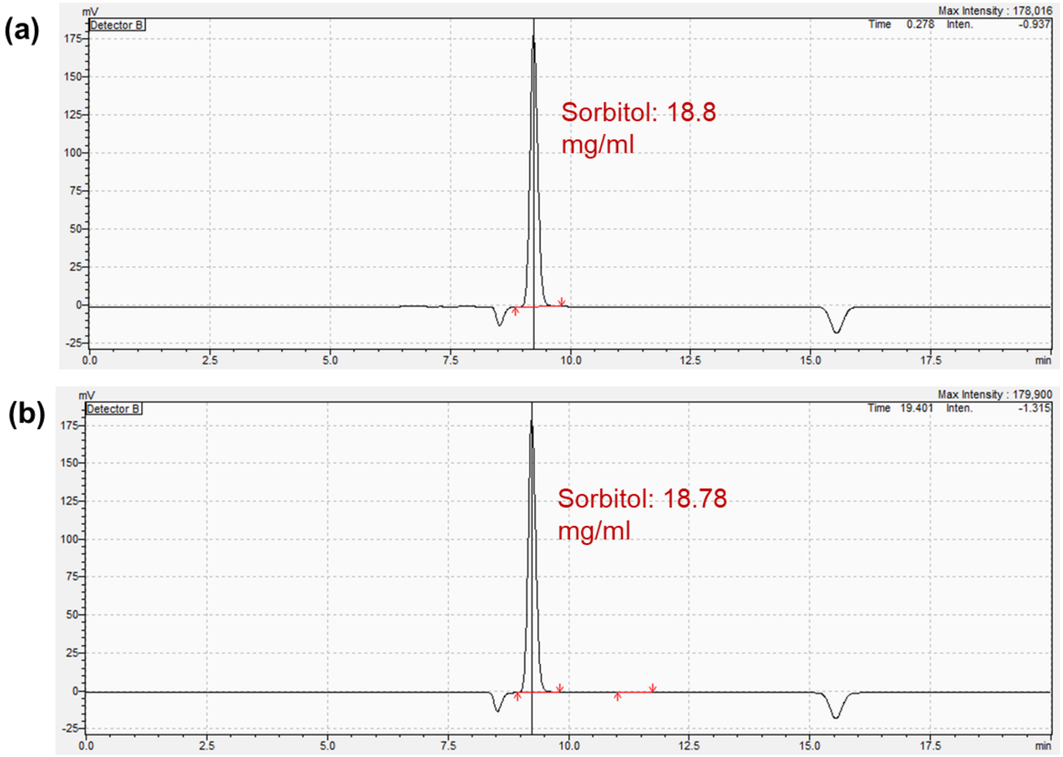

Figure S6. HPLC chromatogram (a) before, and (b) after CO_2RR in the flow cell setup, with CO_2 circulation at 29.4 $mA.cm^{-2}$ current density chrono for 1hr. using 100 mM Sorbitol in the catholyte (0.5M $KHCO_3$). The sorbitol peak appears at 9.23 min.

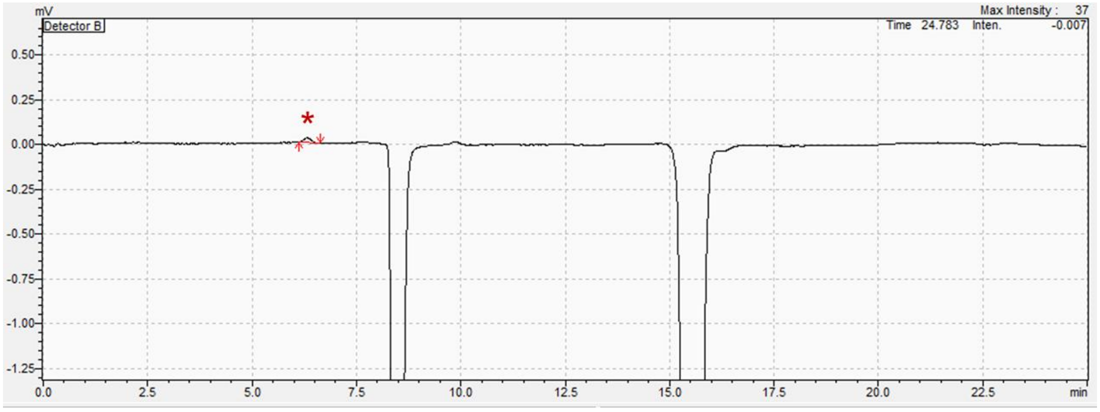

Figure S7. HPLC chromatogram of the pure catholyte (0.5 M $KHCO_3$) obtained using a RID detector, which was employed for the analysis of all liquid products.

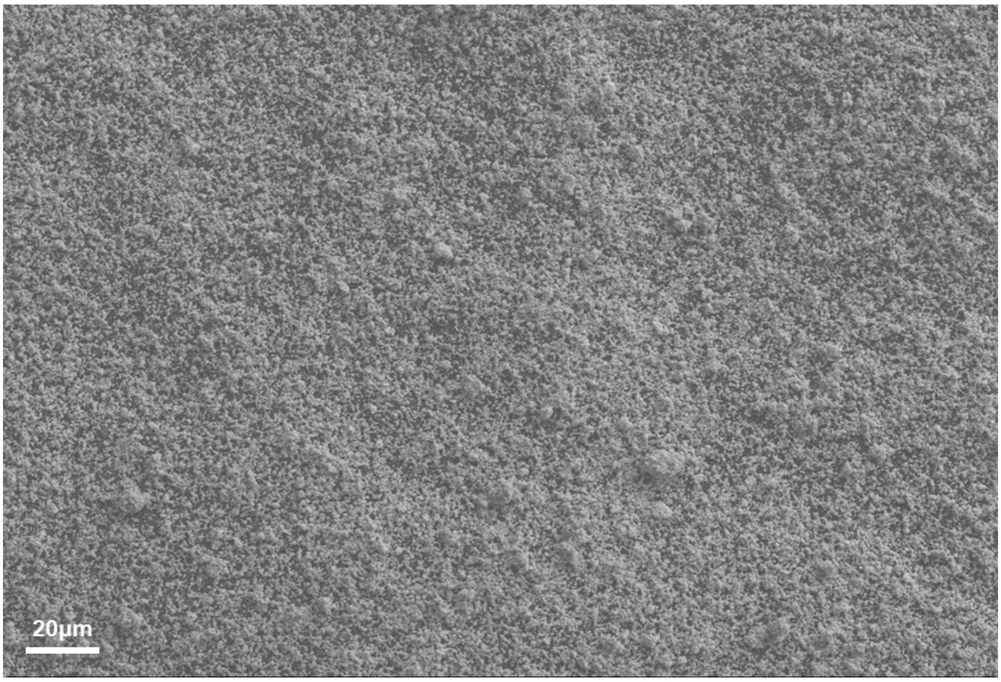

Figure S8. SEM image (Low magnification) of Ag nanoparticles as deposited on the GDE with airbrush (pre – CO_2RR).

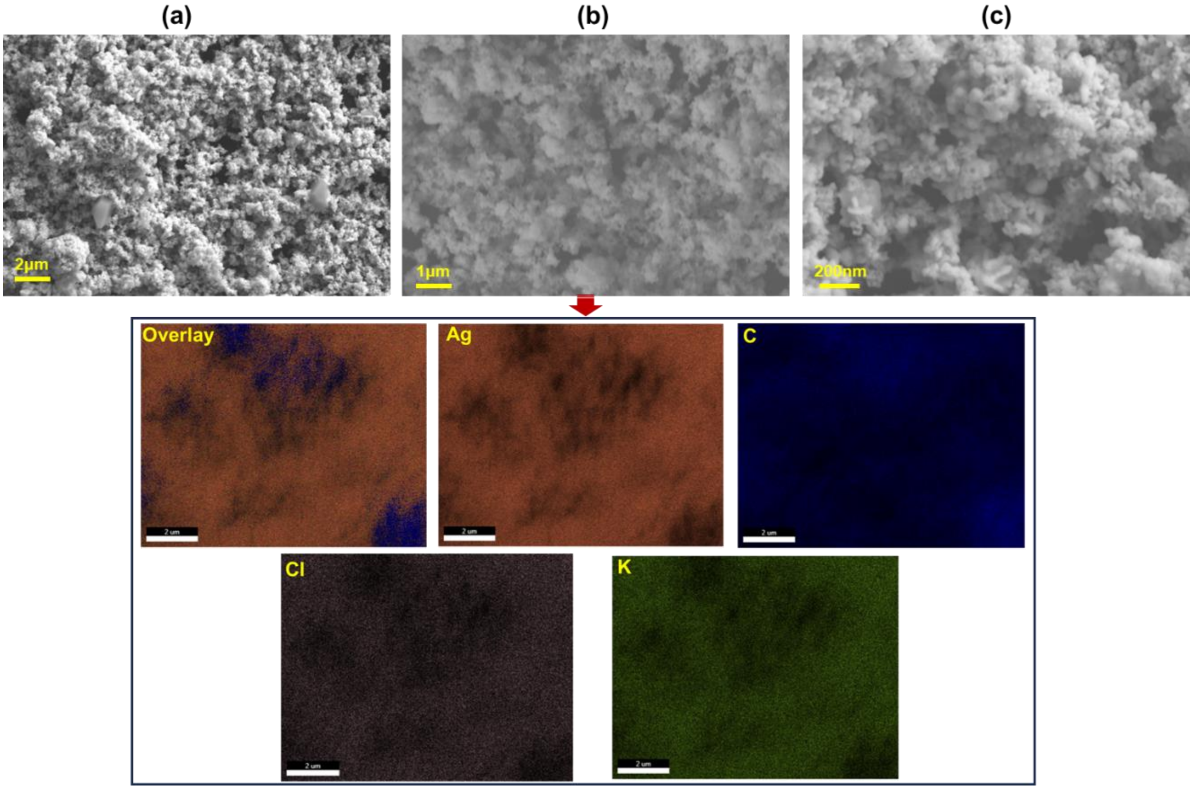

Figure S9. SEM images of the Ag-GDE after CO_2 reduction reaction (post-CO_2RR) with no additive at different magnifications: 1200 fold, (b)10000 fold, and (c) 60000 fold. Corresponding EDX elemental mapping of image (b) showing the distribution of elements.

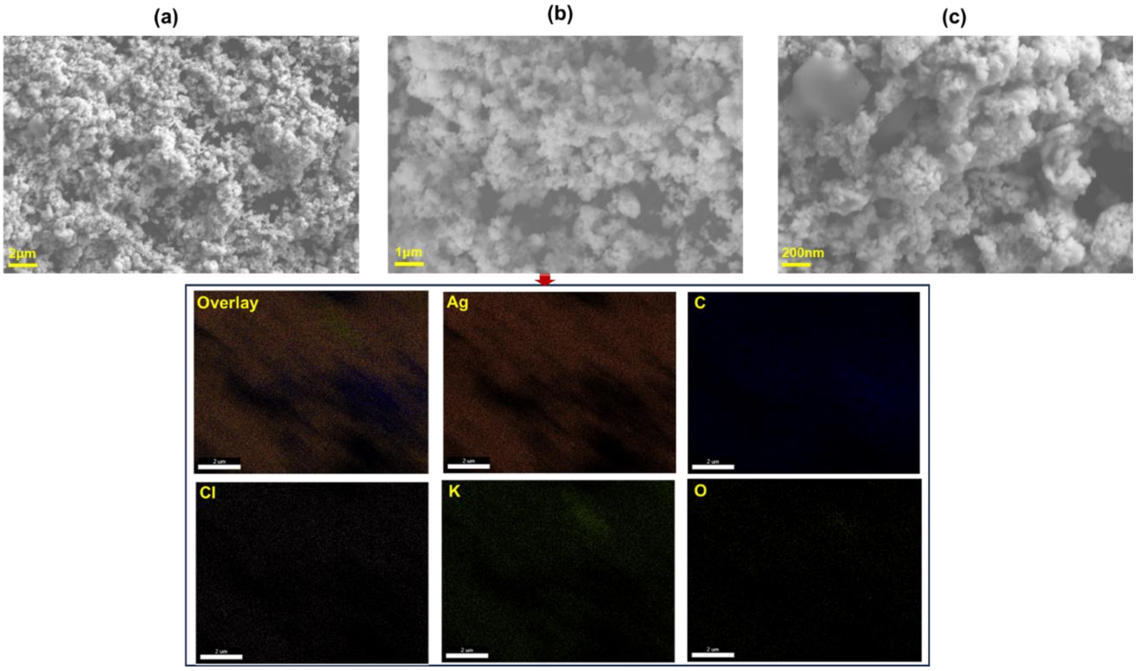

Figure S10. SEM images of the Ag-GDE after CO_2 reduction reaction (post-CO_2RR) with 100mM sorbitol additive at different magnifications: 1200 fold, (b)10000 fold, and (c) 60000 fold. Corresponding EDX elemental mapping of image (b) showing the distribution of elements.

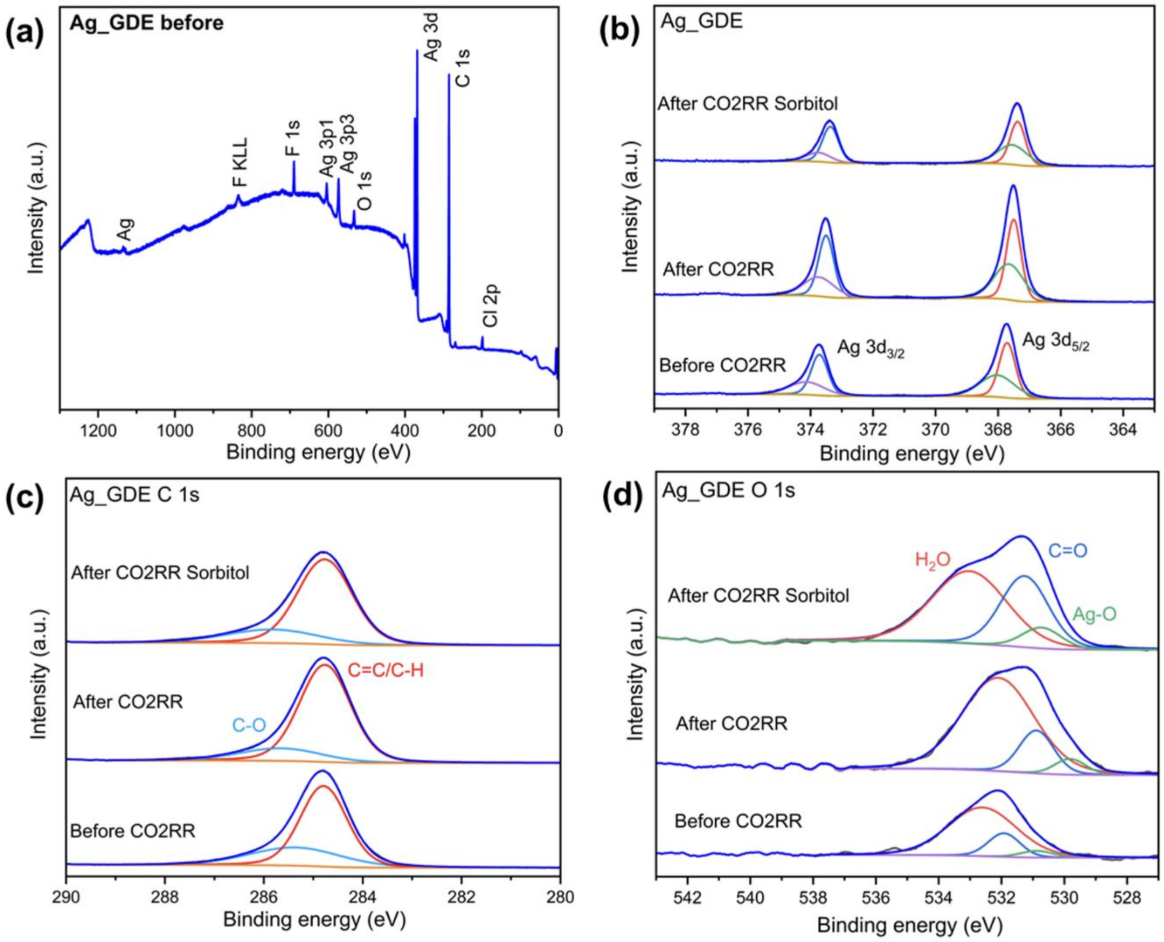

Figure S11. (a) XPS survey of Ag-GDE, (b) XPS spectra of Ag 3d, (c) C 1s and (d) O 1s region for Ag-GDE before and after CO_2RR, with and without sorbitol additive.

MD simulations

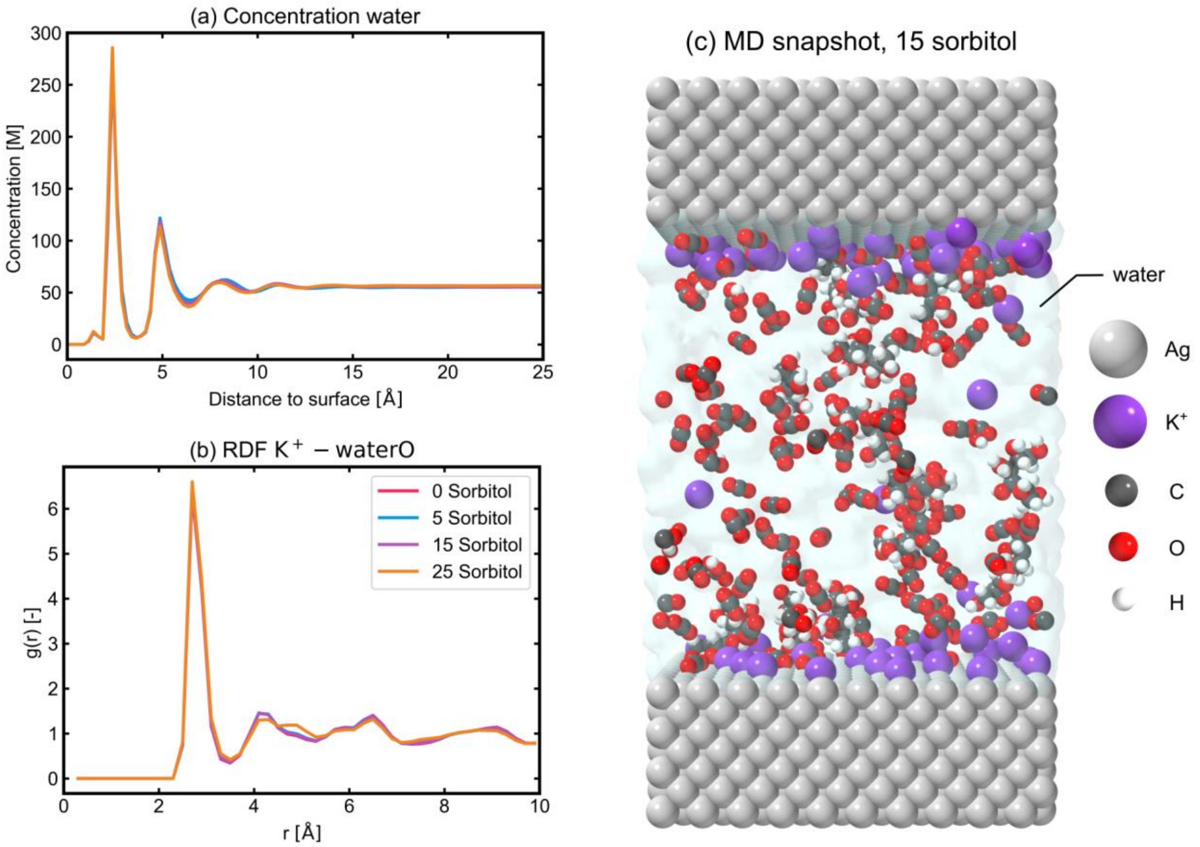

Figure S12. (a) Averaged water density profiles from the electrode surfaces to the bulk region of the confinement; and (b) RDF between K^+ at the interface and oxygen of water for different sorbitol concentrations. (c) Snapshot of MD simulation cell for the case of 15 sorbitol molecules added to the solution.

To simulate the charging of the Ag electrodes, the two innermost layers of the Ag surfaces, each of them consisting of 200 atoms, were assigned partial charges of -0.055 e, resulting in a total negative charge of 44 e. In all simulations 2700 water molecules were used for the solvent, with 20 HCO_3^- molecules added to obtain a bulk electrolyte concentration close to the experimental value. To achieve overall charge neutrality of the system, in total 64 K^+ ions were added

to the solution. The variation of sorbitol concentration was simulated adding 5, 15, and 25 sorbitol molecules, corresponding to a bulk concentration of 0.1, 0.25, and 0.3 M, respectively.

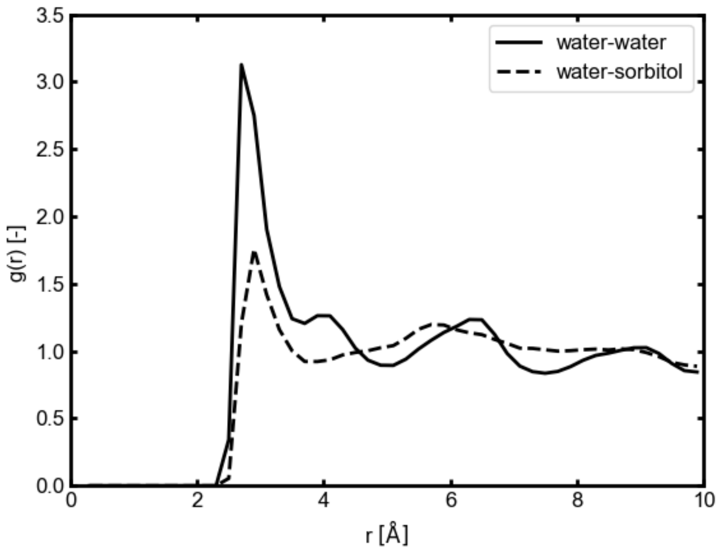

Figure S13. RDFs for the water-water and water-sorbitol interaction (between the oxygen atoms of the respective molecule). Higher RDF peak of water-water indicates a stronger interaction compared to water-sorbitol pair.

MD simulations with partly deprotonated sorbitol

To investigate how high pH environments affect sorbitol molecules, simulations were performed using partially deprotonated sorbitol, where one weaker hydroxyl group was deprotonated. As shown in Figure S13, the density profiles for K^+, CO_2, and water remained consistent across different sorbitol concentrations, aligning with observations from fully protonated scenarios. At the interface, the concentration of negatively charged deprotonated sorbitol decreased due to electrostatic repulsion from the negatively charged silver surface. This decrease in negatively charged sorbitol enhanced the concentration of bicarbonate ions. However, as sorbitol concentration increased, the bicarbonate ion concentration at the interface declined, similar to

trends observed with fully protonated sorbitol. The number of respective molecules at the interface for different sorbitol concentrations is summarized in Table S6.

The RDFs for K^+-water and K^+-CO_2 interactions, depicted in Figure S14(a) and (b), respectively, showed no significant differences compared to cases involving fully protonated sorbitol. While sorbitol presence at the interface promoted the coordination of K^+ with CO_2 (coordination numbers are 0.214, 0.210, 0.235 for 5, 15 and 25 partially deprotonated sorbitol molecules, respectively), the hydration of K^+ by water remained unaffected by changes in sorbitol concentration.

This study underscores that the effects observed in high pH environments are consistent regardless of whether sorbitol is partially or fully protonated, providing a comprehensive understanding of its behavior and interactions at the interface.

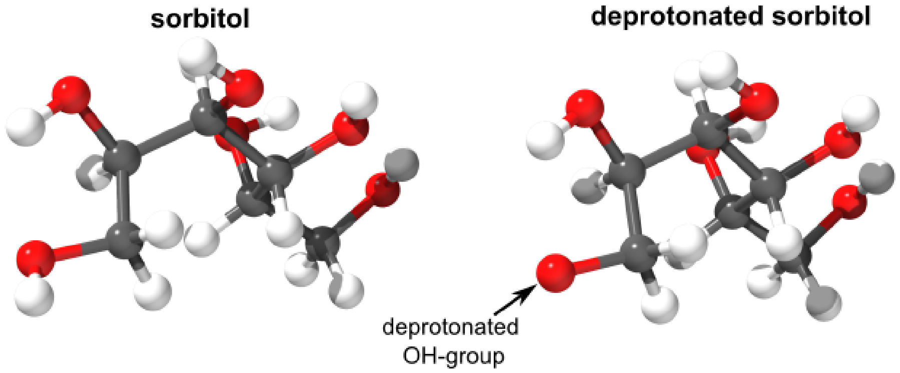

Figure S14. Comparison of the fully protonated and partly deprotonated sorbitol molecule used in the MD simulations.

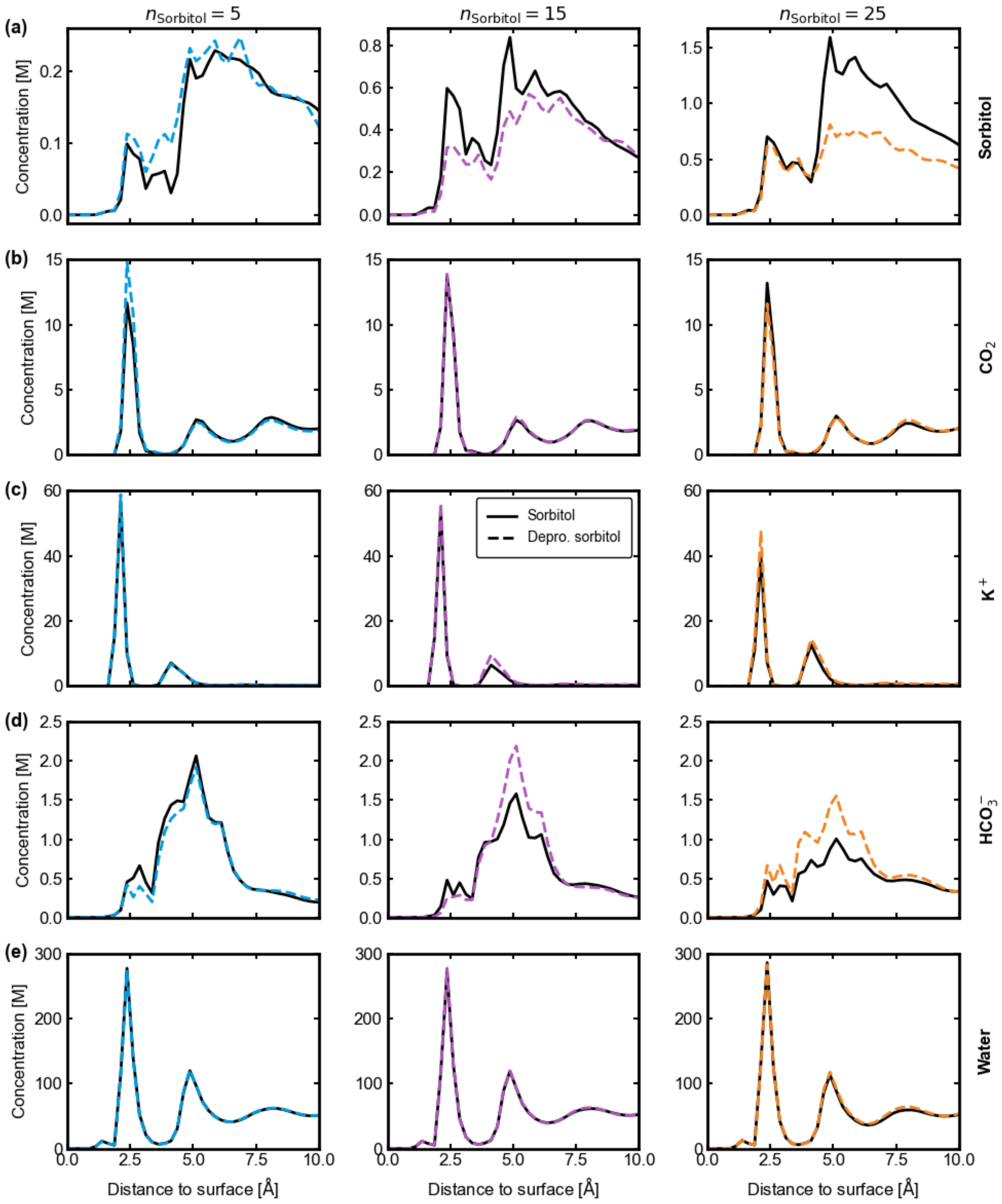

Figure S15. Comparison of averaged concentration profiles for fully protonated and partly deprotonated sorbitol molecules in the vicinity of the Ag surface: (a) sorbitol, (b) CO_2, (c) K^+, (d) HCO_3^-, and (e) water.

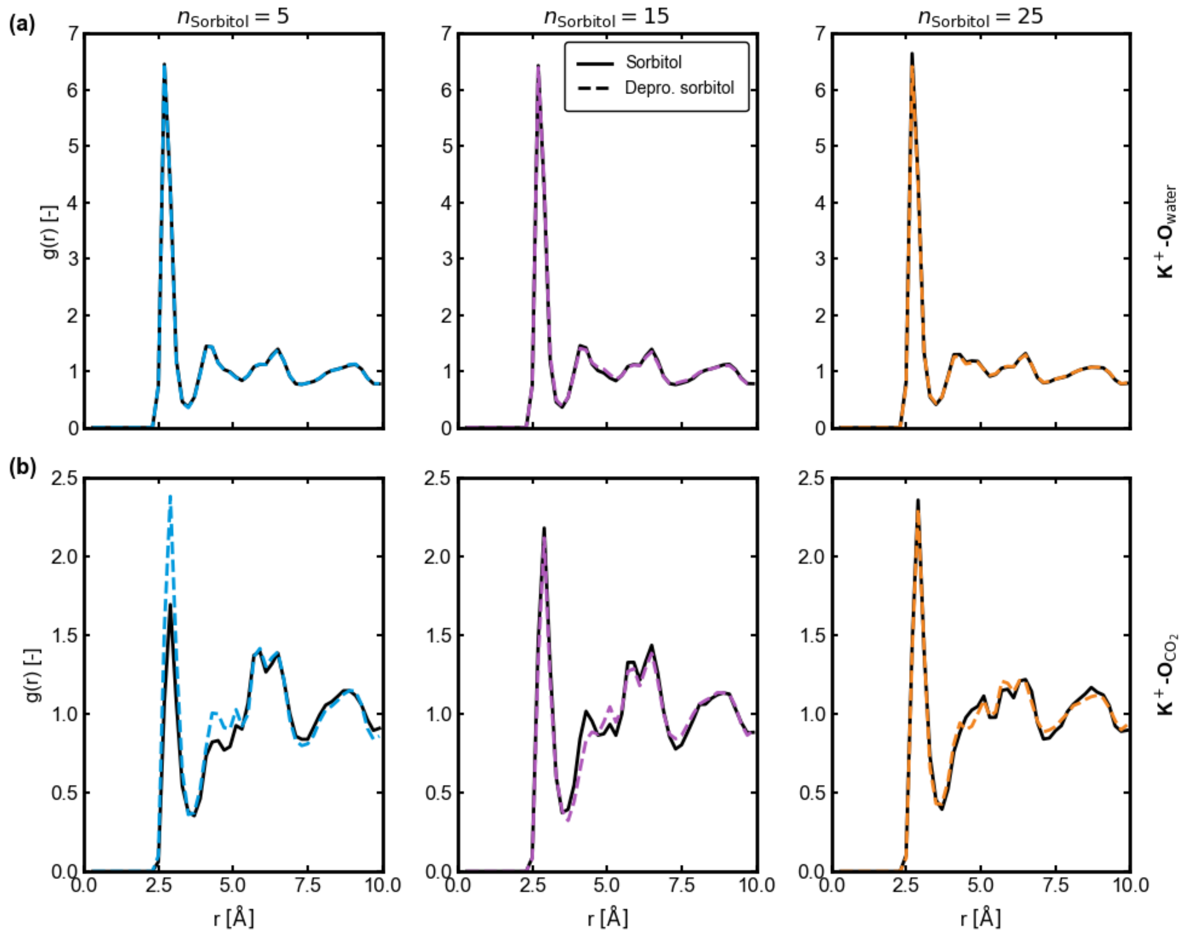

Figure S16. Comparison of the RDFs (a) K^+-O_{water}, and (b) K^+-O_{CO2} for the case of fully protonated and partly deprotonated sorbitol molecules.

Raw data used in the Figures:

Table S1: Figure 1a raw data(Faradaic efficiency of CO_2RR products sorbitol concentration variation from 0 mM to 500 mM at 29.4 mA·cm^{-2} current density)

Sorbitol concentration (mM)	Faradaic efficiency (%)	Error (%)	Faradaic efficiency (%)	Error (%)	Faradaic efficiency (%)	Error (%)	Faradaic efficiency (%)	Error (%)
	H_2		CO		HCOOH		CH_4	
0 mM	10.9	0.8	79.1	1.3	4.5	0.0	0.5	0.3
10 mM	5.9	0.5	84.4	0.4	3.9	0.0	0.3	0.2
100 mM	4.0	0.0	89.6	0.8	5.2	0.0	0.6	0.2
500 mM	2.9	0.3	88.3	4.2	4.4	0.0	0.1	0.2

Table S2: Figure 1b raw data (Faradaic efficiencies comparison between No additive and 100 mM sorbitol additive at different current densities)

Current density (mA.cm^{-2})		Faradaic efficiency (%)	Error (%)	Faradaic efficiency (%)	Error (%)	Faradaic efficiency (%)	Error (%)	Faradaic efficiency (%)	Error (%)
		H_2		CO		HCOOH		CH_4	
9.8	No additive	12.9	0.6	77.7	0.2	1.8	0.0	1.9	0.1
9.8	Sorbitol	8.9	0.6	81.3	0.2	2.9	0.0	2.1	0.1
29.4	No additive	10.9	0.8	79.1	1.3	4.5	0.0	0.5	0.3
29.4	Sorbitol	4.0	0.0	89.6	0.8	5.2	0.0	0.6	0.2
49	No additive	10.7	0.6	81.0	2.7	3.0	0.0	0.2	0.0
49	Sorbitol	4.3	0.0	82.9	0.8	6.0	0.0	0.8	0.2
98	No additive	15.1	2.5	75.6	3.6	4.7	0.0	0.0	0.0
98	Sorbitol	5.4	0.6	79.0	2.7	8.1	0.0	0.8	0.0

Table S3: Figure 1c raw data (H_2/CO ratio (%) without additives and with 100 mM sorbitol at different current densities)

Current density ($mA.cm^{-2}$)	H_2/CO ratio (%)	H_2/CO ratio (%)
	No additive	100mM Sorbitol
9.8	16.6	10.9
29.4	13.8	4.5
49	13.2	5.2
98	20.0	6.8

Table S4: Figure 1d raw data (Partial current density (PCD) of CO and H_2 without additives and with 100 mM sorbitol)

Total Current (A)	PCD CO ($mA.cm^{-2}$)	PCD CO ($mA.cm^{-2}$)	PCD H_2 ($mA.cm^{-2}$)	PCD H_2 ($mA.cm^{-2}$)
	No Additive	100 mM Sorbitol	No Additive	100 mM Sorbitol
0.1	7.6	8.0	1.3	0.9
0.3	23.3	26.4	3.2	1.2
0.5	39.7	40.6	5.2	2.1
1	74.1	77.5	14.8	5.3

Table S5: Figure 2c raw data (FE for the stability of Ag-GDE with 100 mM sorbitol for 10 hours in the flow cell)

time (hr)	CO FE (%) No additive	time (hr)	CO FE (%) 100 mM sorbitol
0.8	87.7	1.7	79.0
1.2	88.0	3.0	79.8
2.0	86.7	3.6	78.8
3.6	85.1	5.2	75.9
4.3	81.9	6.5	69.3

5.1	88.2	8.1	64.6
5.6	89.6	8.9	57.9
6.5	87.7	9.9	54.3
8.4	86.7		
9.3	85.3		
9.9	85.4		

Table S6: Average number of molecules (with standard deviation σ) at the electrode-electrolyte interface (= within the first 6 Å) of the surface, calculated from the concentration/density profiles in Figure 4, S9 and S12.

**Added sorbitol**	**Sorbitol**		**Water**		**CO_2**		**K^+**		**HCO_3^-**	
	**avg.**	**σ**	**avg.**	**σ**	**avg.**	**σ**	**avg.**	**σ**	**avg.**	**σ**
**no sorbitol**										
0	0.00	0.00	221	10.10	7.82	0.81	19.9	0.76	3.47	0.36
**standard sorbitol**										
5	0.37	0.11	223	1.59	6.78	0.82	19.7	0.52	3.35	0.35
15	1.53	0.21	217	2.14	7.41	0.84	18.5	1.02	2.59	0.32
25	2.59	0.29	216	2.99	7.10	1.03	17.8	0.91	1.84	0.20
**deprotonated sorbitol**										
5	0.45	0.17	220	2.28	7.87	0.96	20.1	1.01	2.98	0.38
15	1.07	0.19	219	1.97	7.52	0.7	21.0	1.06	3.06	0.35
25	1.73	0.29	216	3.87	7.1	1.15	17.8	1.26	1.84	0.38
